# Supplementary figures and images for: Bark tissue transcriptome analyses of inverted Populus yunnanensis cuttings reveal the crucial role of plant hormones in response to inversion
Source: PeerJ. 2019 Oct 1;7:e7740. doi: 10.7717/peerj.7740 (PMC6777492; doi:10.7717/peerj.7740)

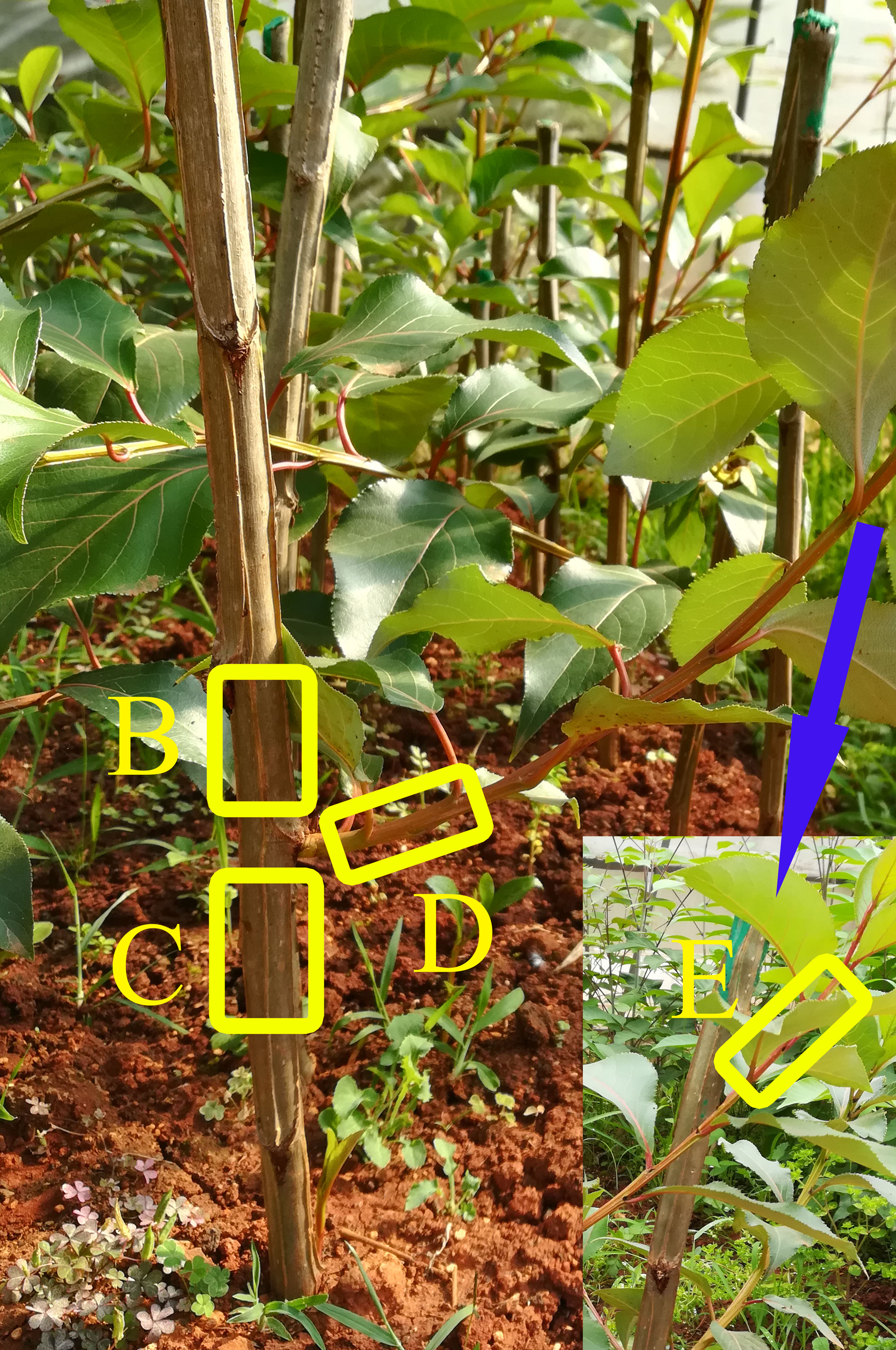

Supplement: Figure S1 [file peerj-07-7740-s001.png]

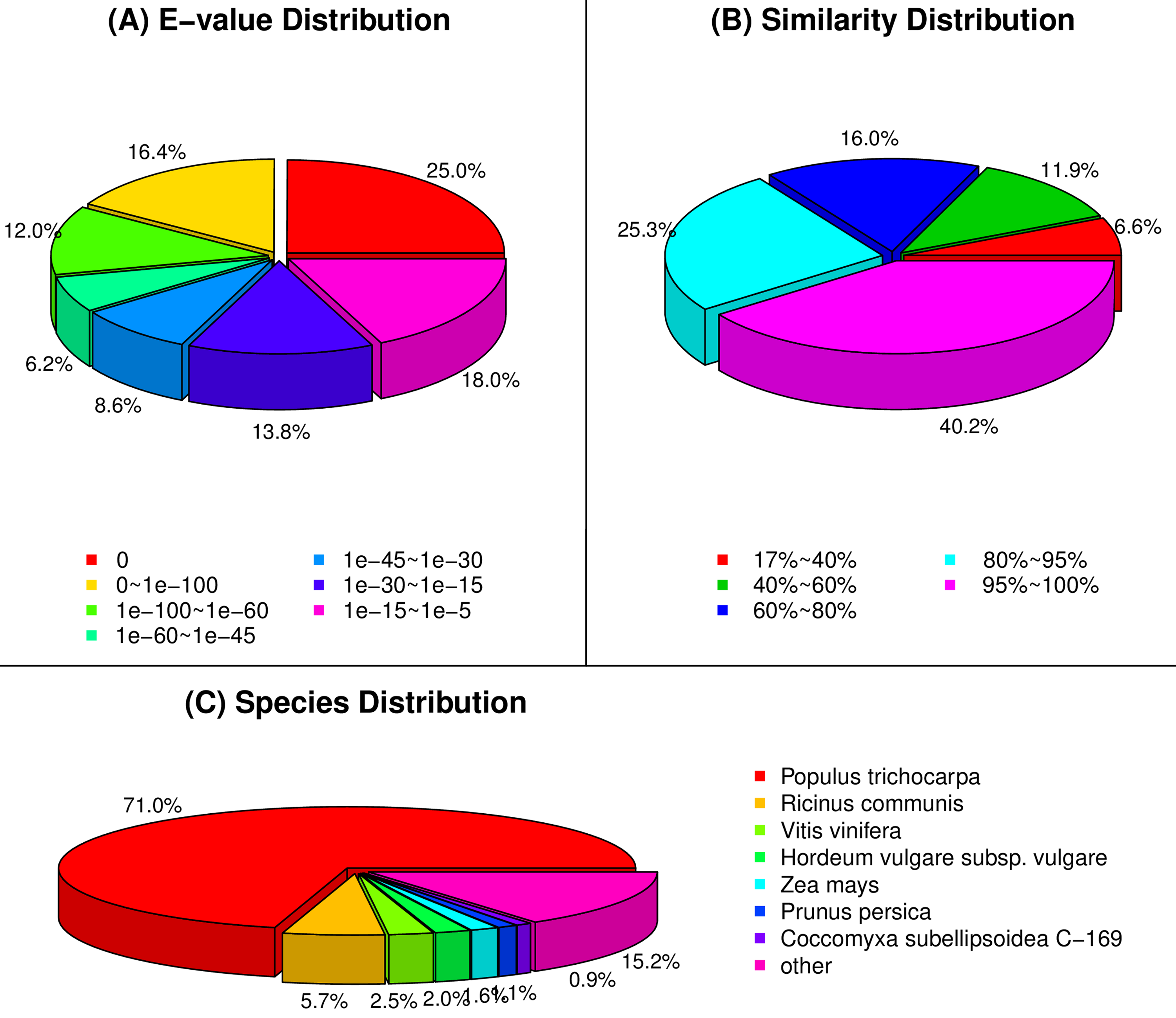

Supplement: Figure S2 — (A) E-value distribution statistics. (B) Similarity distribution statistics. (C) Species distribution statistics. [file peerj-07-7740-s002.png]

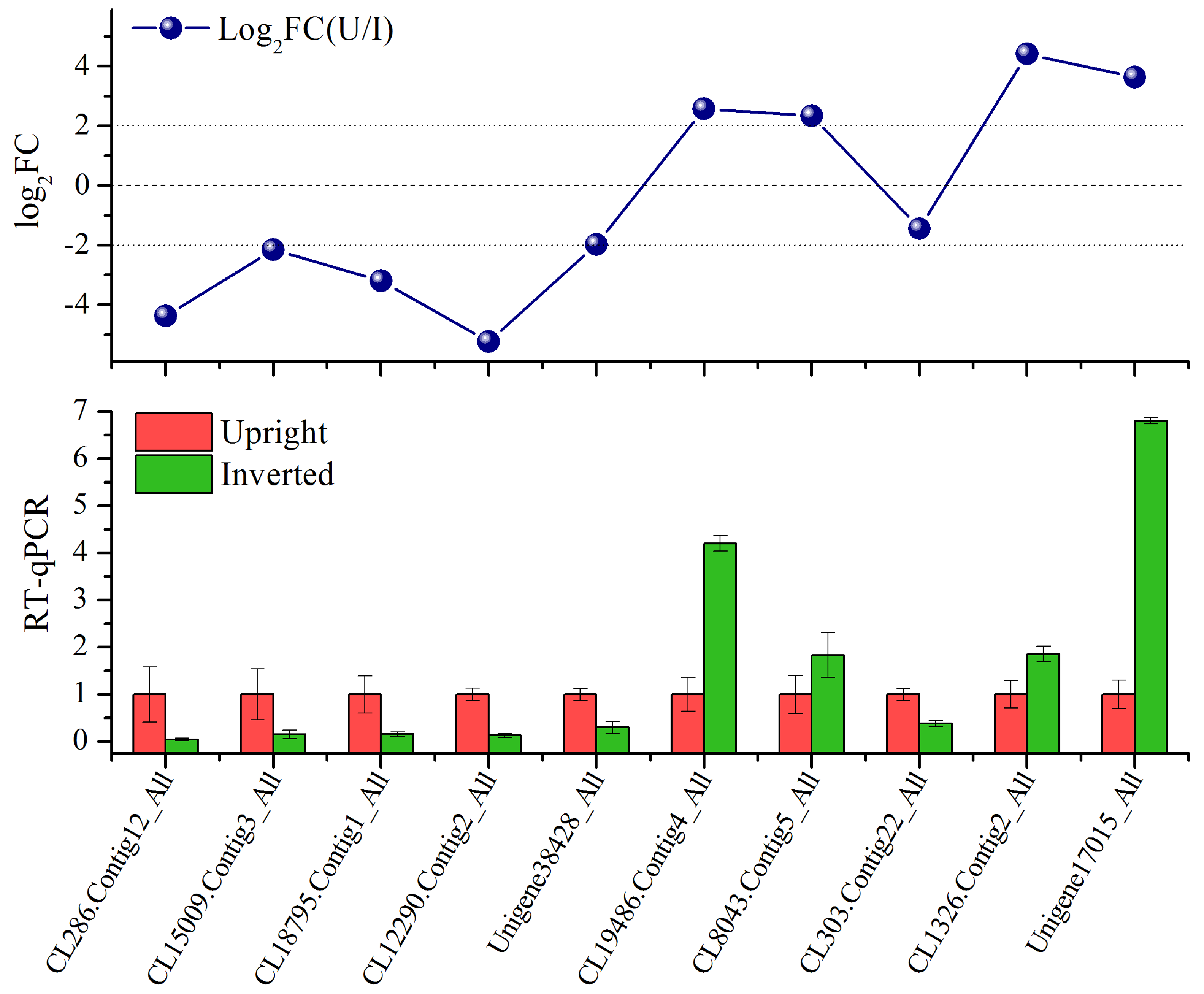

Supplement: Figure S3 — The normalized RT-qPCR data are given as the means ± standard errors (SEs) of three biological replicates. [file peerj-07-7740-s003.png]
