# Supplementary material for: Bark tissue transcriptome analyses of inverted Populus yunnanensis cuttings reveal the crucial role of plant hormones in response to inversion
Source: PeerJ. 2019 Oct 1;7:e7740. doi: 10.7717/peerj.7740 (PMC6777492; doi:10.7717/peerj.7740)
Supplement: Table S1 [file peerj-07-7740-s004.docx]

**Table S1** Parameters of the programs used in *de novo* assembly and functional annotation.

| Program | Parameter |
| --- | --- |
| Trinity v2.3.2 | --seqType fq –min_contig_length 100 –min_glue 3 --group_pairs _distance 250 --path_reinforcement_distance 85 --min_kmer_cov 3 |
| TGICL v2.1 | -l 40 -c 10 -v 20 |
| Blast v2.2.26 | -F F -e 1e-5 |
| Blast2GO v5.0.21 | default |
