# Supplementary material for: Bark tissue transcriptome analyses of inverted Populus yunnanensis cuttings reveal the crucial role of plant hormones in response to inversion
Source: PeerJ. 2019 Oct 1;7:e7740. doi: 10.7717/peerj.7740 (PMC6777492; doi:10.7717/peerj.7740)
Supplement: Table S2 [file peerj-07-7740-s005.docx]

**Table S2** Information on ten selected hub unigenes and endogenous control gene used for RT-qPCR analysis.

| Gene | Group | Forward primer (5'-3') | Reverse primer (5'-3') |
| --- | --- | --- | --- |
| PD-E1 | - | ATGAGAACTGGTGGTATTGGTGC | GTCACAATCTGGGCAGGTTGAAC |
| CL286.Contig12_All | BU | TCCCATCCCACCTGACT | GTGTTGGACCTCCCACTAA |
| CL15009.Contig3_All | BU | GTACTTAGCACCCGAATA | TCCCAACCAGCACAA |
| CL18795.Contig1_All | BU | GGTGCGGCATCTGT | TGGCTGTTTGCTGGT |
| CL12290.Contig2_All | BU | GGAGGCTGATGAGAA | TCGGCGACAAGGA |
| Unigene38428_All | BU | CCAAGCGGAGATAAGG | TGCCCAAACTGTGATG |
| CL19486.Contig4_All | BI | GACGGTGTTGAGGAG | GTTGGAATGTGGGTG |
| CL8043.Contig5_All | BI | GCGGTCGTGGCTAT | CGGACTGCGTTACCT |
| CL303.Contig22_All | CU | TAAATGGTGTCATCCCTC | CTGCTTCCTCGCTAC |
| CL1326.Contig2_All | CI | AGACCAATCCCACCA | TGTAAACCAGTATCCCTC |
| Unigene17015_All | CI | TTGATGGGACCTGG | ATCTTGCGTTGAGTGA |
