# Supplementary material for: Bark tissue transcriptome analyses of inverted Populus yunnanensis cuttings reveal the crucial role of plant hormones in response to inversion
Source: PeerJ. 2019 Oct 1;7:e7740. doi: 10.7717/peerj.7740 (PMC6777492; doi:10.7717/peerj.7740)
Supplement: Table S3 [file peerj-07-7740-s006.docx]

**Table S3** Statistics of sequencing data and assembly results before filtering.

| Sample | Reads | | | | Unigenes | | |
| --- | --- | --- | --- | --- | --- | --- | --- |
|  | Total clean reads (Mb) | Q20 (%) | Q20 (%) | GC (%) | Total Number | Mean length (nt) | N50 |
| BU11 | 46,233,856 | 97.03 | 92.87 | 44.51 | 190,832 | 598 | 1436 |
| BU12 | 41,902,406 | 97.83 | 94.43 | 44.3 | 148,064 | 713 | 1564 |
| BU13 | 44,801,052 | 97.32 | 93.5 | 43.86 | 140,870 | 734 | 1634 |
| BI11 | 42,880,008 | 97.72 | 94.38 | 44.51 | 147,743 | 678 | 1519 |
| BI12 | 41,606,034 | 97.67 | 94.27 | 44.82 | 139,514 | 688 | 1512 |
| BI13 | 40,852,506 | 97.53 | 94.01 | 44.6 | 151,078 | 677 | 1517 |
| CU11 | 39,078,392 | 97.69 | 94.37 | 44.05 | 132,449 | 730 | 1561 |
| CU12 | 44,269,260 | 97.5 | 93.88 | 44.87 | 135,608 | 682 | 1482 |
| CU13 | 40,758,270 | 97.27 | 93.35 | 44.34 | 125,596 | 793 | 1636 |
| CI11 | 42,757,272 | 97.61 | 94.17 | 44.56 | 127,680 | 738 | 1561 |
| CI12 | 41,934,022 | 97.67 | 94.3 | 44.48 | 125,355 | 743 | 1545 |
| CI13 | 46,559,476 | 97.55 | 94.05 | 45.17 | 139,097 | 702 | 1531 |
| DU11 | 49,171,196 | 97.6 | 94.11 | 44.42 | 120,733 | 815 | 1640 |
| DU12 | 47,691,278 | 97.13 | 93.18 | 44.45 | 114,247 | 778 | 1569 |
| DU13 | 45,189,044 | 97.85 | 94.62 | 44.56 | 112,507 | 756 | 1510 |
| DI11 | 44,833,002 | 96.65 | 91.64 | 44.6 | 116,077 | 803 | 1584 |
| DI12 | 43,202,240 | 97.65 | 94.26 | 44.37 | 111,635 | 761 | 1547 |
| DI13 | 40,026,026 | 97.73 | 94.38 | 44.24 | 109,535 | 785 | 1568 |
| EU11 | 46,743,532 | 97.83 | 94.56 | 44.33 | 112,831 | 824 | 1644 |
| EU12 | 44,638,020 | 97.64 | 94.19 | 44.45 | 117,171 | 844 | 1690 |
| EU13 | 46,888,610 | 97.5 | 93.9 | 44.73 | 117,573 | 813 | 1647 |
| EI11 | 47,517,820 | 97.57 | 94.08 | 44.41 | 124,534 | 801 | 1622 |
| EI12 | 45,288,370 | 97.78 | 94.46 | 44.34 | 117,428 | 788 | 1576 |
| EI13 | 44,859,998 | 96.92 | 92.29 | 44.52 | 105,227 | 815 | 1610 |
| All | - | - | - | - | 275,575 | 1223 | 2173 |
