# Supplementary material for: Bark tissue transcriptome analyses of inverted Populus yunnanensis cuttings reveal the crucial role of plant hormones in response to inversion
Source: PeerJ. 2019 Oct 1;7:e7740. doi: 10.7717/peerj.7740 (PMC6777492; doi:10.7717/peerj.7740)
Supplement: Table S5 [file peerj-07-7740-s008.docx]

**Table S5** RT-qPCR verification of the transcriptome results.

| Hub unigenes | Transcriptome | RT-qPCR | | | |
| --- | --- | --- | --- | --- | --- |
|  | log_2_FC(Inversion/Upright) | Upright | Standard error | Inversion | Standard error |
| CL286.Contig12_All | -4.37241 | 1 | 0.585156 | 0.046671 | 0.021251 |
| CL15009.Contig3_All | -2.14679 | 1 | 0.539778 | 0.154753 | 0.09031 |
| CL18795.Contig1_All | -3.19597 | 1 | 0.393678 | 0.16079 | 0.045007 |
| CL12290.Contig2_All | -5.23527 | 1 | 0.13195 | 0.132071 | 0.046271 |
| Unigene38428_All | -1.97854 | 1 | 0.12709 | 0.302683 | 0.124964 |
| CL19486.Contig4_All | 2.572684 | 1 | 0.362721 | 4.20507 | 0.16484 |
| CL8043.Contig5_All | 2.338666 | 1 | 0.404303 | 1.83679 | 0.471845 |
| CL303.Contig22_All | -1.45124 | 1 | 0.12709 | 0.380073 | 0.064741 |
| CL1326.Contig2_All | 4.414505 | 1 | 0.289911 | 1.855536 | 0.163489 |
| Unigene17015_All | 3.632325 | 1 | 0.299288 | 6.806825 | 0.066723 |
